# Nuclear and mitochondrial data reveal different evolutionary processes in the Lake Tanganyika cichlid genus *Tropheus*

**DOI:** 10.1186/1471-2148-7-137

**Published:** 2007-08-14

**Authors:** Bernd Egger, Stephan Koblmüller, Christian Sturmbauer, Kristina M Sefc

**Affiliations:** 1 Department of Zoology, University of Graz, Universitätsplatz 2, 8010 Graz, Austria

## Abstract

**Background:**

Cichlid fishes are notorious for their wealth of intra- and interspecific colour pattern diversity. In Lake Tanganyika, the endemic genus *Tropheus *represents the most impressive example for geographic variation in the pattern and hue of integument colouration, but the taxonomy of the over 100 mostly allopatric colour morphs remains to a large degree unresolved. Previous studies of mitochondrial DNA sequence data revealed polyphyly of the six nominally described species and complex phylogeographic patterns influenced by lake level fluctuations and population admixture, and suggested the parallel evolution of similar colour patterns in divergent evolutionary lineages. A gene tree of a rapidly radiating group may be subject to incomplete and stochastic lineage sorting, and to overcome this problem we used multi-locus, nuclear AFLP data in comparison with mtDNA sequences to study diversification, migration and introgression in *Tropheus *colour morphs in Lake Tanganyika.

**Results:**

Significant incongruence between phylogenetic reconstructions from mitochondrial and AFLP data suggested incomplete sorting of mitochondrial haplotypes as well as frequent introgression between differentiated lineages. In contrast to the mitochondrial phylogeny, the AFLP phenogram was largely congruent with species classifications, colour pattern similarities, and in many cases also with the current geographic distribution of populations, and did not produce evidence of convergent colour pattern evolution. Homoplasy in the AFLP data was used to identify populations that were strongly affected by introgression.

**Conclusion:**

Different evolutionary processes were distinguished by the combination of mitochondrial and AFLP data. Mitochondrial phylogeographic patterns retained signals of large-scale migration events triggered by historical, major lake level fluctuations, whereas AFLP data indicated genetic cohesion among local groups of populations resulting from secondary contact of adjacent populations in the course of the more frequently occurring, minor lake level fluctuations. There was no support for the parallel evolution of similar colour patterns in the AFLP data. Genetic signatures of introgression and hybridisation detected in several populations suggest that lake level fluctuations drove the stunning diversification of *Tropheus *morphs not only through population fragmentation, but also by promoting hybridisation between differentiated morphs in secondary contact.

## Background

Incongruence among gene trees is a widespread phenomenon in phylogenetic studies of recently and rapidly radiating species [1-7]. Mitochondrial DNA markers have several evolutionary properties that make them suitable for phylogenetic and phylogeographic studies, but inferences within and between closely related species can be misled by introgression and incomplete lineage sorting [2,8-10]. In recent years, the amplified fragment length polymorphism technique (AFLP, [11]) has found application in evolutionary studies to resolve phylogenies both at the intraspecific population level as well as between several million year old lineages [12-16]. In cichlids and other rapidly diversifying taxa, where speciation events often precede fixation of alleles within a species (e.g. [9]), the combined analysis of numerous AFLP markers recently has been shown to overcome the problems of incomplete sorting of alleles between speciation events [12,17-21].

The tribe Tropheini comprises a highly diverse assemblage of cichlid fishes endemic to Lake Tanganyika that radiated from a riverine ancestor and evolved into the most abundant group in the rocky littoral zone [22,23]. As a sister group to the Tropheini, the remaining "modern haplochromines" (sensu [23]) also originated in Lake Tanganyika and later dispersed through the rivers of eastern Africa to colonise the younger lake basins of Lakes Malawi and Victoria [23]. The genus *Tropheus *represents the most impressive example for the phenomenon of geographic variation in body colour pattern in Lake Tanganyika's cichlid species, and over 100 mostly allopatric colour variants are recognised [24,25]. Due to their diversity, *Tropheus *represent a promising system for studies of colour pattern evolution, and for the same reason, specimens of the genus have become extremely popular among aquarists. So far, six nominal species have been described based on morphological characters (*T. moorii *BOULENGER, 1898; *T. annectens *BOULENGER, 1900; *T. duboisi *MARLIER, 1959; *T. brichardi *NELISSEN and THYS VAN DEN AUDENAERDE, 1975; *T. kasabae *NELISSEN, 1977 and *T. polli *AXELROD, 1977; [26]). However, there are no scientific data on the distribution of the species around the lake, and many *Tropheus *populations from different regions of the lake cannot be unequivocally identified to species [26]. Due to the uncertainties in the species-level taxonomy of the genus and the high level of colour pattern diversity on small geographic scales, populations are generally identified by their locality rather than by species (e.g. [27]), and this practice is also adopted in the present work. The popularity of *Tropheus *recently prompted attempts to improve the taxonomic coverage of the genus, which were presented in the popular literature but have not been validated by scientific studies. For example, in addition to the scientifically described nominal species, Konings [24] suggested five taxa (*T. *sp. "black", *T. *sp. "red", *T. *sp. "Ikola", *T. *sp. "Mpimbwe") and proposed assignments of populations to the different species based on phenotypic traits such as body shape and coloration. Schupke [25] described more than 100 *Tropheus *populations and arranged them into 13 colour lineages.

The evolution and phylogeographic history of the genus was investigated in four studies that were based on mitochondrial sequence data and revealed highly complex patterns of lineage distribution [27-30]. One concurrent finding of these studies was the paraphyletic status of the genus, as *T. duboisi *represents a distinct unit, which is not in a sister group relationship to the remaining species of the genus *Tropheus*. Sturmbauer et al. [27] defined 12 mitochondrial lineages based on a phylogenetic reconstruction using a parsimony method implemented in TCS [31], which have evolved in the course of two major radiations about 700–945 Ka and 399–540 Ka. Both diversification events could be correlated to extreme changes of lake level, and a lowstand of Lake Tanganyika in the recent past (11–17 Ka; [32]) might have shaped the present distribution of lineages.

**Figure 1:**
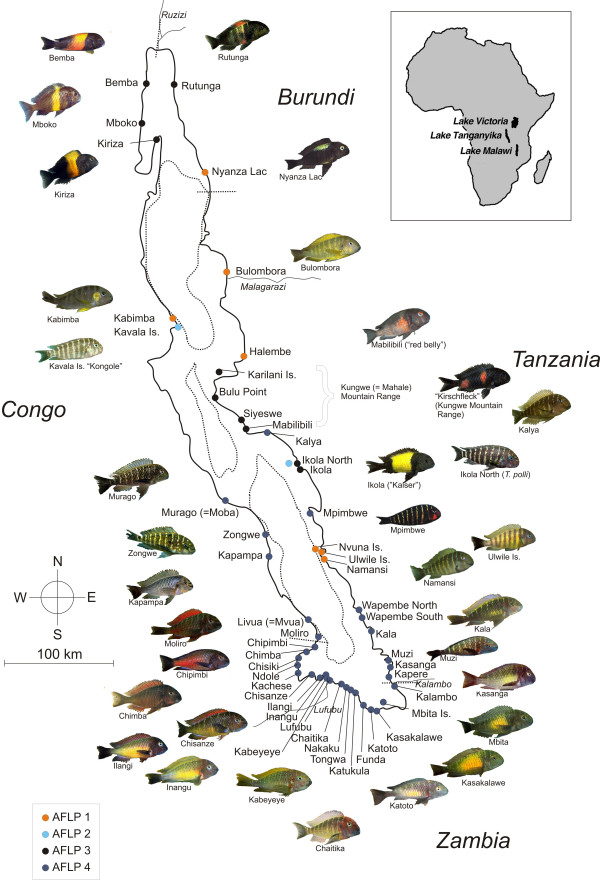
**Map of Lake Tanganyika**. Map of Lake Tanganyika showing sampling sites and examples of the colour morph diversity of *Tropheus*. Deepwater basins of the lake were drawn with dotted lines. Rivers names are printed in italics. Photographs of fish were labelled by the locality where the respective morphs occur, with additional designations used in the aquarium trade added in quotation marks. Photographs were kindly provided by Peter Berger [62]. The dots marking sampling sites were colour-coded to show the distribution of the four major AFLP clades.

Lake level fluctuations displace benthic populations and entail cycles of population admixture and isolation, and indeed, several *Tropheus *populations comprise a mixture of different mitochondrial lineages, indicating introgression during secondary admixis of formerly differentiated populations. Mitochondrial haplotype sharing across opposite shorelines further testifies that lineages were able to cross the lake along the ridges between the lake basins during periods of low lake level [27]. Despite the historically unstable population distribution along the shore, *Tropheus *populations display high levels of genetic structure across very short geographic distances in both mitochondrial and nuclear markers ([33] and unpublished data). Gradual among-population differences in colour pattern generally increase with geographic distance between populations, and major habitat barriers (like sandy bays) often separate phenotypically highly distinct morphs (see Fig. 1 for a map of Lake Tanganyika and *Tropheus *colour morphs). However, populations displaying similar colour patterns are not always closely related in terms of mtDNA phylogenetic reconstructions, which suggests introgression among lineages and convergent evolution of colour patterns in the genus. Only recently, hybridisation has been proposed as an important factor for the generation of diversity in animals [7], and recent phylogenetic studies showed that introgression has occurred between distinct lineages within the cichlid species flocks and even might have given rise to hybrid speciation in some cases [6,18,34-36]. Some of the colour pattern diversity in *Tropheus *may well be due to the introgression events indicated by mitochondrial data.

To overcome the limitations of phylogenetic inference from a single gene, both AFLP and mtDNA sequence data were collected from *Tropheus *populations around Lake Tanganyika. Mitochondrial and nuclear-multilocus tree reconstructions were tested for consistency, and hypotheses based on previous mtDNA studies, such as introgression patterns and convergent evolution of certain colour patterns, were evaluated with respect to the AFLP data. The different information provided by the two marker systems was used to address different processes affecting the evolutionary history and current phylogeography, such as large-scale migration and hybridisation between colour morphs. Due to the uncertainty of species-level taxonomy in *Tropheus*, we apply Harrison's [37] definition of hybridisation as "the interbreeding of individuals from two populations, or groups of populations, which are distinguishable on the basis of one or more heritable characters". Finally, the molecular data were compared to current species assignments and to the suggested classifications of Konings [24] and Schupke [25].

## Results

### Mitochondrial sequence relationships

**Figure 2:**
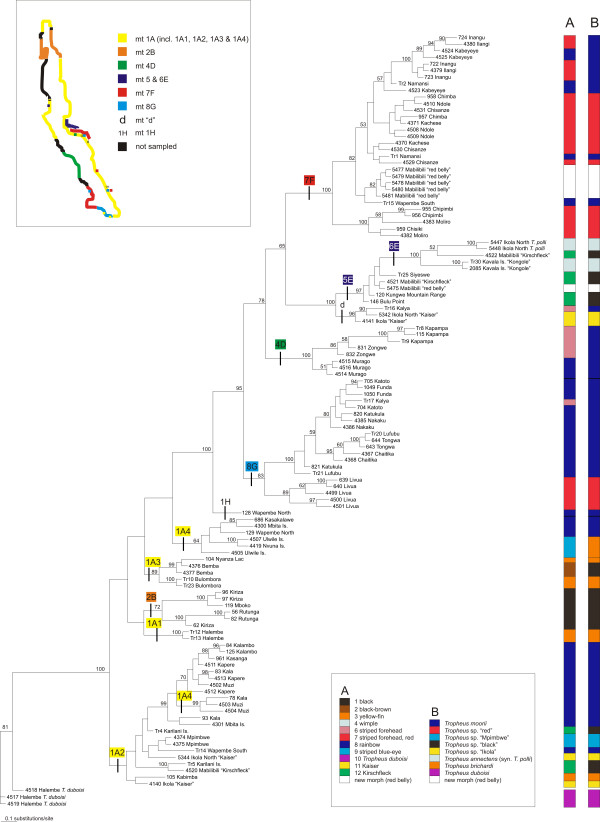
**mtDNA tree**. Bayesian inference tree based on the mitochondrial control region sequences. The mtDNA-lineages defined by Sturmbauer et al. [27] are indicated above branches, and their approximate distribution around the lake (adapted from [27]) is shown in the map (top left). Posterior probability values are shown near the respective nodes. The bars on the right-hand side show the assignment of the samples to (A) colour lineages [25] and (B) to the species classification suggested by Konings [24]. Same-coloured bar sections spanning paraphyletic clades are divided by thin black lines to indicate the inconsistency with the tree topology.

The 117 samples included in this study represent 9 of the 12 previously defined mitochondrial lineages based on parsimony network clusters reconstructed in the program TCS (Fig. 2; [27]). The topologies of the trees derived by Bayesian inference (Fig. 2, 117 samples) and a NJ analysis (117 samples; [see Additional file 1]) were consistent with the NJ topology inferred in a comprehensive analysis of 462 *Tropheus *samples [27]. Differences between tree topologies concern nodes with little support from posterior probability and bootstrap values. Fifty-nine of the samples presented here were not included in the dataset of Sturmbauer et al. [27] and a description of their positions in the phylogenetic reconstructions is presented as supplementary information [see Additional file 2].

As previously observed (e.g. [27,28]), the mitochondrial phylogenetic reconstruction of *Tropheus *populations was in several instances inconsistent with the geographic distribution of populations on the one hand and with colour pattern similarities among populations on the other hand. Figure 2 shows that there is little agreement between mitochondrial sequence relationships and suggested species assignments [24] and colour lineage classifications [25].

### AFLP genotype clusters

AFLP data were obtained with ten primer combinations and consisted of 914 fragments (146 constant, 687 parsimony informative and 81 parsimony-uninformative sites). The resulting tree topology was markedly different from the mtDNA-based reconstruction. Shimodeira-Hasegawa [38] LRT found the difference of 904 likelihood units between mtDNA and AFLP topologies to be significant (P < 0.05).

**Figure 3:**
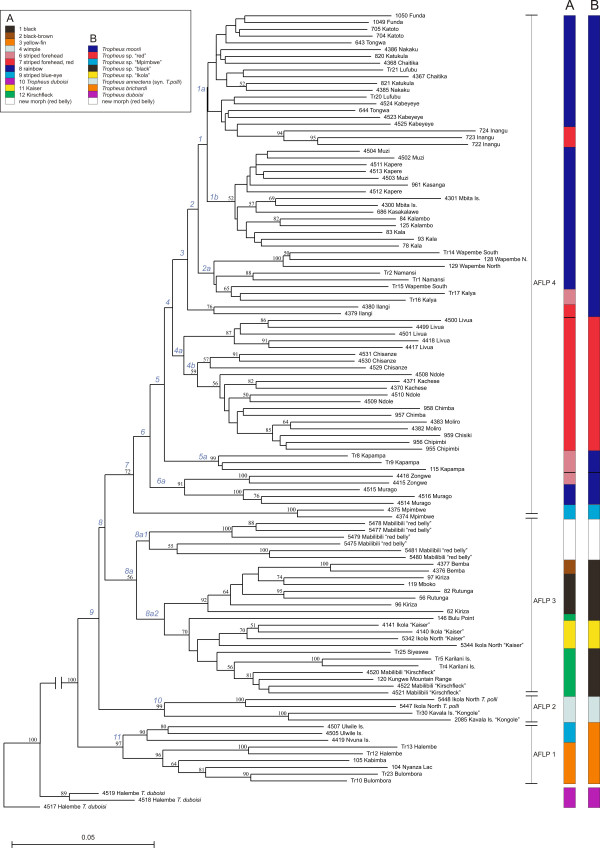
**AFLP tree**. Neighbour-joining tree based on AFLP genotype distances. Bootstrap support of more than 50% is shown in black near the respective nodes. The long branch connecting the outgroup was truncated. Nodes examined by the homoplasy excess test (Fig. 4) are labelled in light-blue. The four well-supported AFLP clades are delineated on the right-hand side, and the coloured bars depict the assignment of the samples to (A) colour lineages [25] and (B) to the species classification suggested by Konings [24]. Same-coloured bar sections spanning paraphyletic clades are divided by thin black lines to indicate the inconsistency with the tree topology. The geographic distribution of the AFLP clades is shown in Fig. 1.

Unlike the mitochondrial sequence phylogeny, the AFLP phenogram revealed clusters of phenotypically and, in part, geographically close populations (Fig. 1, Fig. 3). Four major clades could be identified: The basal clade (AFLP 1) comprised a heterogeneous group of samples from geographically quite distant localities, whose populations have been assigned to *T. brichardi *(northern basin: Nyanza Lac, Bulombora, Kabimba and Halembe, southern basin: Ulwile Island, Namansi and Nvuna Island). Clade AFLP 2 contained *T. polli *sampled from Ikola at the eastern shore of the central basin as well as *T. *"Kongole" from Kavala Island at the southwestern shoreline of the northern basin, which phenotypically closely resemble *T*. *polli*. Clade AFLP 3 comprised the colour morphs displaying a dark body colour with a conspicuous yellow or red band or with blotches – e.g. the popular *T. *"Kaiser" and "Kirschfleck" – as well as the newly described morph "red belly" from Mabilibili with a less contrast rich colour pattern. Within this clade, the populations from Kiriza, Mboko, Bemba and Rutunga in the north, and from Ikola and the Kungwe mountain range in the east were grouped in two sister sub-clades consistent with their geographic distribution. Finally, clade AFLP 4 unites the *T. moorii *populations from the southern basin of the lake with *Tropheus *from Mpimbwe splitting at the base. Within AFLP 4, the *T. moorii *populations were grouped according to their geographic range and phenotypic conformity (Fig. 1), but statistical support for these subclades was weak. In more detail, the red-coloured morphs from Chisanze to Livua grouped together in one subclade. The colour morphs with a yellow blotch on their body side (Kasakalawe to Kala) were grouped as a subclade sister to the colour morphs displaying a bluish body colour (Katukula to Chaitika). The population in Katoto is apparently an admixture of both morphs [33]. "Yellowish" morphs from Lufubu, Kabeyeye and Inangu were placed in the "bluish" subclade, whereas the yellow Ilangi morph was resolved as sister group to the clade that unites "bluish" and "yellow-blotch" morphs with more "red-brownish" populations from Wapembe and Namansi and from Kalya further north.

Samples from the same population always belonged to the same major AFLP clade even when they were not grouped next to each other, whereas they were often distributed across several mtDNA lineages. Suggested species assignments [24] and colour lineage classifications [25] by and large agreed with AFLP clades (Fig. 3; see Discussion).

### Signals of introgression and hybridisation: AFLP homoplasy excess test and mitochondrial relationships

Hybrid genotypes contain AFLP-fragments from both parental clades, which appear as homoplasies and reduce bootstrap support for the parental clades [7]. Hence, removal of hybrid taxa from the phylogenetic analysis will increase bootstrap values for the parental clades significantly beyond any changes in bootstrap support induced by removal of any other non-hybrid taxa. Such changes in bootstrap support caused by the removal of any random clades could, for example, be due to retained ancestral polymorphism in the data set. The procedure of removing various different clades from the analysis therefore serves to distinguish signals of hybridization from signals of ancestral polymorphism. Note that ancestral polymorphism, introgression involving several clades as well as homoplastic mutations will prevent bootstrap support values for a parental clade from reaching 100% even when the hybrid taxon is removed from the data set. Furthermore, it is theoretically possible that a "hybrid" signal in the AFLP data is solely due to retained ancestral polymorphism, when drift was much weaker in some populations than in others.

**Figure 4:**
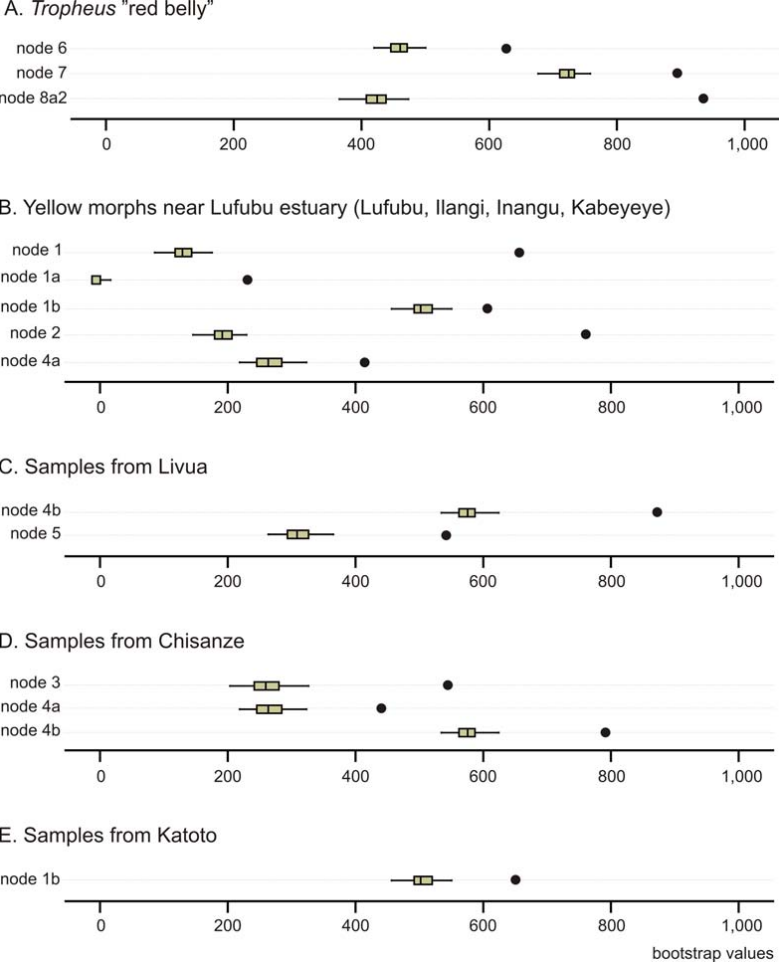
**Homoplasy excess test**. Potential hybrid and introgressed taxa identified by the AFLP homoplasy excess test. The boxplots illustrate the distributions of bootstrap support values obtained in 53 taxon-reduced bootstrap analyses for nodes, which experienced a significant increase in bootstrap support (marked by solid circles) upon removal of the following taxa: A, *Tropheus *"red belly"; B, the yellow coloured morphs near the Lufubu River estuary (samples from Lufubu, Ilangi, Inangu and Kabeyeye); C, samples from Livua; D, samples from Chisanze; E, samples from Katoto. The nodes are identified in Fig. 3.

We removed each of the 51 population samples at a time, and calculated bootstrap support for 15 nodes in the taxon reduced data set. Additionally, in two instances, several populations were removed at once (samples of the four yellow coloured morphs from Ilangi, Inangu, Kabeyeye and Lufubu; and samples of *T. polli *from Ikola North together with *T. *"Kongole" from Kavala Island). The homoplasy tests identified a number of potentially introgressed populations, which is in line with the interpretation of the published mtDNA based phylogeographic data claiming that very few populations experienced longer periods of independent evolutionary history. This made reconstruction of possible hybrid parents very difficult in most cases, as parental populations of hybrid taxa were probably often themselves affected by introgression. Figure 4 shows boxplot diagrams of the distribution of bootstrap values obtained from the 53 taxon-reduced runs for selected examples, which were interpreted as possible hybrid origin of or substantial introgression into the taxon whose removal caused the significant increase in bootstrap support of a node. See Figure 3 for positions of the nodes mentioned in the following text. The geographic distribution, mitochondrial lineage assignment and phenotype of the populations involved in the here described examples of introgression is given as supplementary information [see Additional file 2].

Removal of the *Tropheus *"red belly" individuals resulted in a rise of the bootstrap value at node 8a2 from 418 to 948 (Fig. 3 and 4A), which strengthens support for nuclear genetic cohesion of this – phenotypically plausible – group. Our data suggest that the "red bellies" represent a hybrid morph with one parent in the AFLP-3 clade – likely a *T*. "Kirschfleck" –, but the AFLP data failed to pinpoint the second candidate parent clade. Removal of the yellow coloured morphs sampled near the Lufubu estuary (Fig. 4B) increased the bootstrap values at node 1 ("bluish" + "yellow-blotched", from 154 to 666), node 1a ("bluish", from 14 to 240), node 2 (from 219 to 776) and node 4a ("red", from 266 to 427), and slightly raised support at node 1b ("yellow-blotched", from 518 to 621). These homoplasy signals together with mitochondrial haplotype composition of the "yellow" morphs [see Additional file 2] suggest ancient introgression between the "red" and "bluish" morphs. The removal of individuals from Livua (Fig. 4C) resulted in a rise of bootstrap values at nodes 4b ("red" morphs, from 588 to 885) and 5 (from 205 to 554), and removal of Chisanze samples from the AFLP dataset (Fig. 4D) had strong effects on the bootstrap values of node 4a ("red", from 266 to 453), node 4b ("red" without Livua, from 588 to 800) and node 3 (306 to 557). Both cases are suggestive of introgression between distinct lineages. The population at Katoto, which is located near a bay that separates "bluish"and "yellow-blotched" morphs, has been assumed to originate from an admixture of the two morphs, since it displays an intermediate colour pattern and contains haplotypes from both sides of the bay [33]. In the AFLP analysis, the removal of the two samples from Katoto caused a slight increase of the bootstrap value at node 1b (the "yellow-blotched" clade) from 518 to 665 (Fig. 4E), which was beyond the bootstrap value fluctuations caused by removal of other taxa. Although the signal is weak, it is consistent with introgression of the "yellow-blotched" morph into the Katoto population.

There was no significant increase in bootstrap support for nodes 5a, 8, 8a, 10 and 11 in any of the taxon-reduced runs.

## Discussion

Our results demonstrate a high degree of incongruence between mtDNA derived and nuclear based phylogenetic reconstructions, suggesting incomplete lineage sorting and introgression in the course of the evolution of the genus *Tropheus*. Alternatively, sex-biased dispersal behaviour could have contributed to the observed disparities. However, congruent genetic structure was detected in mitochondrial haplotype and nuclear microsatellite data in a population genetic analysis (unpublished data), which indicates that, at least on small geographic scales, both sexes show concordant dispersal behaviour. Incomplete lineage sorting is not uncommon in the rapidly diversifying lacustrine cichlids of East Africa, and was, for example, demonstrated in phylogenetic studies on Lake Victoria cichlids [39] and the Mbuna cichlids of Lake Malawi [12,40-42]. The importance of incomplete lineage sorting and/or hybridisation has also been discussed for Lake Malawi non-Mbuna cichlids [43] and the Lake Tanganyika cichlid species assemblage [9,44], in particular with respect to the tribes Eretmodini [34], Lamprologini [6,36,45,46] and Perissodini [47]. Given the rapidity of the mitochondrial lineage diversification in the genus *Tropheus *in combination with population displacements and admixtures triggered by lake level fluctuations [27], it is reasonable to expect signals of both incomplete lineage sorting and introgression in the genetic data.

The phylogeographic distribution of the mitochondrial haplotypes of *Tropheus *populations is highly complex. Distribution ranges of mitochondrial lineages overlap in several regions of the lake and distinct mtDNA lineages often comprise populations from geographically very distant localities and with distinct phenotypes (Fig. 2; see also [27]). Additionally, mitochondrial sequence relationships are much at odds with suggested species delineations in the scientific as well as in the aquarist literature [24] and with a proposed classification of the genus into lineages based on colour pattern similarities ([25]; Fig. 2). In contrast, the phenogram based on AFLP data (Fig. 3) is largely congruent with species classifications (e.g. *T. brichardi *in clade AFLP 1, *T. polli *in clade AFLP 2), colour pattern similarities (AFLP 3 and sub-clades in AFLP 4), and in many cases also with the current geographic distribution of populations (sub-clades in AFLP 3; AFLP 4 and sub-clades therein; Fig. 3). Besides the recently discovered "red belly", the morphs included in clade AFLP 3 were classified as *T. *sp. "black" and *T. *sp. "ikola" (consisting of the "Kaiser" morph from Ikola) by Konings [24], and Schupke [25] elaborated an even more fine-scale subdivision of this clade into four colour lineages. Mitochondrial sequences of these morphs were resolved in different lineages, and suggested convergent evolution of the conspicuous colour pattern. In contrast, the AFLP data indicate monophyletic ancestry of the geographically disjunct, but phenotypically similar populations around Bemba in northern Lake Tanganyika, and along the Kungwe mountain range in the central lake basin, and furthermore signal genetic cohesion between the similarly coloured populations within each region (see Fig. 1 and 3). The separate taxonomic placement of the "Kaiser" morph from Ikola outside *T. *sp. "black" is not supported by our data.

Within clade AFLP 4, there was no bootstrap support for the relationships between subclades. This lack of AFLP-based resolution may reflect a high frequency of introgression among the populations inhabiting the shallow southern tip of Lake Tanganyika. Here, even rather minor lake level fluctuations must have displaced littoral populations, which were forced to follow the shifting shoreline, and thus induced admixture of several previously isolated populations. Likewise, the populations from the shallow northern tip of the lake (samples from Kiriza, Mboko, Bemba and Rutunga in AFLP 3) were not resolved by locality within their clade, probably due to the same reason. Additionally, sampling sites along the southern shoreline were more densely distributed than in other regions, and signals of introgression between neighbouring populations were therefore picked up more readily. Nevertheless, although significant bootstrap support is lacking, the grouping of the southern populations within clade AFLP 4 is consistent with the geographic distribution and the phenotypic similarities, and by and large identifies the "red" morphs, the "bluish" morphs and the "yellow-blotched" morphs as differentiated units with genetic connectivity within colour morphs. Still, the distinction of *T*. sp. "red" from *T. moorii *[24] is not fully supported by our data.

In contrast to the continuous geographic distribution of clade AFLP 4 containing the southern populations, the three other clades have a fragmented distribution along the lakeshore, which follows from the disjunct distributions of the species joined into these clades [24]. The processes leading to the patchy distributions of these species remain puzzling, but may be consequences of colonisation waves following lake level fluctuations and competitive interactions between differentiated colour morphs and species [27].

Despite the disparities between mtDNA and AFLP data, both marker types supply valuable and complementary phylogenetic and phylogeographic information. Whereas AFLP data for the first time demonstrated the genetic cohesion of some *Tropheus *taxa, the mitochondrial data provided details about the colonisation history of the genus *Tropheus *and revealed multiple waves of diversification and dispersal (see also [27]). For example, mitochondrial data give evidence of migration across the lake or into distant populations on the same lakeshore, where the introgressed mtDNA haplotypes occasionally persisted and sometimes even drifted to high frequencies [27]. In the multi-locus AFLP data, the signal of individual migration events was lost through recombination, whereas the genetic continuity between neighboring populations and the common ancestry of phenotypically discernible clades becomes visible. Large-scale migrations are probably associated with isolated periods of severe lake level lowstands [27]. Similarly, the gene flow between adjacent populations along a stretch of shoreline indicated by AFLP data is likely to occur in a punctuated fashion, when occasional drops of the lake level impose secondary contact, rather than by ongoing dispersal, as populations have been shown to be genetically differentiated at distances of less than 10 km [33].

In recent studies of cichlids, hybridisation has been advanced as an additional pathway for the generation of novel taxa [6,7,18,35,46], and hybrids have been observed in nature [e.g. [36,48,49]]. Usually, hybrids are first recognised by their intermediate colour patterns or body shape, but in the case of *Tropheus*, the great wealth of intergradient colour morphs complicates a phenotypic detection of potential hybrids. The AFLP data revealed numerous potential cases of hybridisation and introgression between morphs, which may have given rise to novel morphs in some instances. Positive colour-assortative mating between highly distinct *Tropheus *morphs in secondary contact has recently been demonstrated in a population consisting of artificially admixed morphs, but reproductive isolation was weak between less distinct morphs [50], and similar results were obtained in laboratory mate choice experiments (Egger et al. unpublished data). Under natural conditions, genetic evidence for the admixture of morphs displaying distinct colour patterns exists in the population of Katoto, while examples for the maintenance of a reproductive barrier between very distinct phenotypes is found in the sympatric *T. *sp. "black" and *T. polli *from Ikola North, and in the sympatric occurrence of "Kirschfleck"-morphs with *T. polli *and *T. brichardi *in different regions of the lake. Hybridisation between very distinct morphs could also have been promoted by turbid water conditions such as in river estuaries or caused by strong rainfalls, like it is known for Lake Victoria cichlids [48]. There is some evidence in our data that the yellow morphs near the Lufubu River estuary are strongly influenced by high levels of introgression from the neighboring "bluish" and "red" morphs or even may have originated from hybridisation between these morphs. In laboratory experiments, "bluish" and "red" morphs mate assortatively (Egger et al. unpublished data), but variables such as visibility and resource distribution may have considerable impact on mate choice in the field.

## Conclusion

We conclude that the combination of mitochondrial and AFLP data provides valuable information on different processes affecting the evolutionary history of *Tropheus*. Mitochondrial phylogeographic patterns retained signals of large-scale migration events triggered by major lake level fluctuations, whereas the genetic cohesion among local groups of populations indicated by AFLP data results from gene flow between adjacent populations brought about by relatively frequent minor lake level fluctuations. Introgression and hybridisation between differentiated populations, morphs and species was evident in both types of molecular data, and we suggest that lake level fluctuations drove the stunning diversification of *Tropheus *morphs not only through population fragmentation, but also through hybridisation between differentiated morphs in secondary contact.

## Methods

### Taxon sampling and DNA extraction

We obtained genetic data from 117 *Tropheus *individuals originating from 51 localities, covering almost the entire shoreline of Lake Tanganyika. Denser sampling along the southern shoreline reflects the political situation in the countries bordering the lake (see Fig. 1). Fish were either collected in the field or purchased from ornamental fish importers. Samples and sampling localities are given in Figure 1 and a supplementary table [see Additional file 3]. DNA was isolated from ethanol-preserved fin-clips or white muscle tissue using proteinase K digestion followed by a high-salt extraction technique [51]. DNA concentration was measured using the Fluoroskan Ascent FL-System. All samples were adjusted to a concentration of 60 ng/μl.

### mtDNA sequence analyses

Previous studies of mitochondrial phylogeography in *Tropheus *by Sturmbauer and Meyer [28], Sturmbauer et al. [30], Baric et al. [29] and Sturmbauer et al. [27] have accumulated 5' partial control region sequence data for more than 450 *Tropheus *samples. Fifty-eight of these published sequences were used in this study [see Additional file 3], and additional sequences from 59 individuals were obtained as follows. Polymerase chain reaction (PCR), purification of PCR products, and sequencing followed the protocol described in Duftner et al. [52]. The primers used for PCR and sequencing were L-Pro-F [53] and TDK-D [54]. DNA fragments were purified with Sephadex™ G-50 (Amersham Biosciences) following the manufacturer's instruction and visualized on a 3130 × l capillary sequencer (Applied Biosystems).

DNA sequences were aligned by eye using the Sequence Navigator software (ver. 1.0.1 Applied Biosystems). The alignment comprised 367 bp, with 243 constant sites, 27 parsimony-uninformative characters and 97 parsimony-informative characters. Empirical base frequencies in this data set were A = 0.3540, C = 0.2206, G = 0.1308, T = 0.2946. Phylogenetic relationships among the 117 samples used in the present study were estimated by a Bayesian method of phylogenetic inference using MrBayes (ver. 3.0b4 [55]), applying the substitution model HKY, as suggested by Modeltest (ver. 3.06 [56]), with *T. duboisi *as outgroup. Posterior probabilities were obtained from a 4,000,000 generation Metropolis-coupled Markov chain Monte Carlo simulation (ten chains; chain temperature, 0.2) with parameters estimated from the data set. Trees were sampled every hundred generations and the first 20,000 trees were excluded as burn-in to allow likelihood values to reach stationary. The resulting tree (Fig. 2) serves to represent the mitochondrial structure and diversity of *Tropheus *populations as resolved in Sturmbauer et al. [27]. Additionally, NJ was run in PAUP* (ver. 4.0b10, [57]) under the HKY+I+G [58] substitution model as estimated on the data by Modeltest (invariable sites, 0.47, gamma shape parameter, 0.90), with *T. duboisi *as outgroup.

Previous work [27] combined mtDNA control region sequences of 462 *Tropheus *samples into a neighbour joining (NJ) tree, and classified samples into mitochondrial lineages corresponding to unconnected networks resolved by a phylogenetic reconstruction using a parsimony method implemented in TCS (ver. 1.13 [31]). The phylogenetic analysis of the 117 samples included in the present work was conducted in order to enable a comparison between AFLP data and the documented mitochondrial diversity of *Tropheus*, and to illustrate references to the mtDNA phylogeography published in [27].

### AFLP analysis

AFLP genotypes were obtained from the same 117 individuals as used for the mitochondrial analysis, except for four samples from Zongwe and Livua, which had to be replaced by alternative samples from the same locations, because the DNA previously used for mtDNA sequencing was of insufficient quality for AFLP genotyping. Restriction digestion of 60 ng of genomic DNA was performed in a total volume of 50 μl using 0.5 μl *Mse*I (10 units/μl, New England Biolabs), 0.25 μl *EcoR*I (200 units/μl, New England Biolabs), 5 μl enzyme buffer (10×), 0.5 μl BSA (100×) and high performance liquid chromatography (HPLC) water, and incubated for three hours at 37°C. For adaptor ligation, 1 μl *EcoR*I-adaptor (50 pmol/μl), 1 μl *Mse*I-adaptor (5 pmol/μl), 1 μl T4 ligase buffer, 0.2 μl T4 DNA ligase and 6.8 μl HPLC water were added to the product of the restriction digestion and incubated over night at 22°C. The ligation products were subsequently diluted to 180 μl using HPLC water. The preselective amplifications contained 3 μl of the diluted ligation product, 0.4 μl each of *EcoR*I and *Mse*I preselective primers (10 μM), 2 μl 10× MgCl_2 _buffer, 2 μl 10× dNTP mix (10 μM) and 0.6 μl *Taq *DNA polymerase (5 units/μl, BioTherm™) in a final volume of 20 μl. The preselective primers consisted of the adaptor primer sequence with a single selective nucleotide at the 3' end (*EcoRI*-pre: A, *MseI*-pre: C). The preselective PCR used the following temperature profile: 2 min at 72°C followed by 20 cycles of 20 sec at 94°C, 30 sec at 56°C, and 2 min at 72°C, then a holding step at 60°C for 30 min. PCR products were diluted 1:10 for selective amplification. For selective amplifications we used the following ten primer combinations: *EcoR*I-ACA/*Mse*I-CAA, *EcoR*I-ACA/*Mse*I-CAG, *EcoR*I-ACA/*Mse*I-CAC, *EcoR*I-ACA/*Mse*I-CAT, *EcoR*I-ACT/*Mse*I-CAT, *EcoR*I-ACT/*Mse*I-CAA, *EcoR*I-ACT/*Mse*I-CAG, *EcoR*I-ACT/*Mse*I-CAC, *EcoR*I-ACG/*Mse*I-CTG, *EcoR*I-ACT/*Mse*I-CTG. The PCR cocktail contained 1 μl of the diluted preselective PCR product, 6.3 μl HPLC water, 0.8 μl 10× dNTP mix (10 μM), 1 μl 10× MgCl_2 _buffer, 0.4 μl *Taq *DNA polymerase (5 units/μl; BioTherm™), 1 μl selective *Mse*I primer (10 μM) and 1 μl selective *EcoR*I primer (1 μM) labeled with the fluorescent dye FAM. The temperature profile for the selective PCR was as follows: 2 min at 94°C followed by 10 cycles with 20 sec at 94°C, 30 sec at annealing temperature, which decreased in each cycle by 1°C from 65°C to 56°C, and 2 min at 72°C. The PCR continued for 25 cycles with 20 sec at 94°C, 30 sec at 56°C, and 2 min at 72°C, followed by a holding step at 60°C for 30 min. All amplifications were performed on a GeneAmp PCR system 9700 (Applied Biosystems). Selective amplification products were visualized on an ABI 3130 × l automated sequencer (Applied Biosystems) along with an internal size standard (GeneScan-500 ROX, Applied Biosystems).

Raw fragment data were analyzed using Genemapper (ver. 3.7, Applied Biosystems). Automated analysis with a fluorescent threshold value set to 40 units yielded 2694 fragments, but the results were compromised by the fact that the restriction and ligation reactions had been carried out in two batches, as ten DNA samples became available only when the first batch of samples had already been processed. We detected a small number of fragments that were fixed in one batch of samples but missing from the other, which resulted in the clustering of those individuals that had been processed together. The second restriction and ligation batch also contained 6 replicate samples that had already been processed in the first set of reactions, which allowed us to identify and exclude the artifact, batch-specific fragments. Consequently, the dataset does not include bands with fixed presence in one batch of samples and fixed absence in the other batch. Furthermore, we found automated scoring problematic with respect to fragments that show gradual intensity differences between samples, as in such cases, any detection threshold must be arbitrary. Consequently, presence and absence of peaks were scored by eye in the Genemapper software within a range of 100–500 bp, and only distinct, major fragments were considered and assembled as a binary (1/0) matrix. In a few cases, fragments were scored as missing data when character states could not be determined unambiguously. Manual scoring yielded a lower number of marker bands than automated scoring, but data quality was substantially improved by this approach. Manually scored genotypes of replicates matched to a level of 96%. The resulting data matrix consisted of 914 fragments in 117 samples.

The program Treecon (ver. 1.3b [59]) was used to construct a NJ tree from a pairwise distance matrix [60]. As with mtDNA analyses, three samples of *T. duboisi *were used as outgroup taxa. Bootstrap values from 1000 pseudo-replicates were calculated to assess support for the reconstructed tree topology. There is no explicit consensus in the literature with regard to the most appropriate tree reconstruction method for AFLP data. With the present data set, the use of a different distance estimator [61] for NJ analysis and tree reconstruction by parsimony criteria yielded the same major clades as the here presented tree.

Differences between the mtDNA and AFLP tree topologies were statistically evaluated using the Shimodeira-Hasegawa [38] likelihood ratio test as implemented in PAUP* (ver. 4.0b10, [57]).

### Testing for homoplasy excess

A tree-based method [7] was performed to test for homoplasy excess introduced by hybridisation and introgression. Theoretically, the inclusion of a hybrid taxon into a multi-locus phylogeny introduces homoplasy with clades that include the hybrid parents. Removal of the hybrid taxon should therefore decrease the amount of homoplasy in the dataset and increase bootstrap support for clades containing hybrid parents or their descendents. Conversely, removal of non-hybrid taxa should not affect the bootstrap support of other nodes. One population sample at a time was removed from the data, and bootstrap support for 15 nodes in the AFLP tree (see Fig. 3) was computed. This procedure yielded a distribution of bootstrap values for each of the 15 nodes, which was analysed by the method of boxplots. Values more than 3 interquartile ranges away from the box were considered significant outliers.

## Authors' contributions

All authors were involved in sampling. The study was designed by KMS. AFLP data were collected and analysed by BE. Mitochondrial sequence data were collected and analysed by SK. The manuscript was drafted by BE and KMS with contributions by SK and CS. All authors read and approved the final version.

## Supplementary Material

Additional file 1 NJ tree. NJ tree based on the mitochondrial control region sequences. Click here for file

Additional file 2 Detailed information on mitochondrial sequence relationships and signals of introgression and hybridisation. The file provides additional, detailed information on mitochondrial sequence relationships and signals of introgression and hybridisation. Click here for file

Additional file 3 List of samples used for mtDNA and AFLP analyses. The file provides a list of samples used for mtDNA and AFLP analyses, with information about sequence accession numbers, coordinates of sampling sites, and assignment of samples to species and colour lineages. Click here for file
