# Supplementary material for: Nuclear and mitochondrial data reveal different evolutionary processes in the Lake Tanganyika cichlid genus Tropheus
Source: BMC Evol Biol. 2007 Aug 14;7:137. doi: 10.1186/1471-2148-7-137 (PMC2000897; doi:10.1186/1471-2148-7-137)

Additional file 1 - NJ tree

Neighbour-joining tree based on the mitochondrial control region sequences. The mtDNA-lineages defined by Sturmbauer et al. [27] are indicated above branches. Bootstrap support of more than 50% is shown near the respective nodes. The bars on the right-hand side show the assignment of the samples to (A) colour lineages [25] and (B) to the species classification suggested by Konings [24]. Same-coloured bar sections spanning paraphyletic clades are divided by thin black lines to indicate the inconsistency with the tree topology.

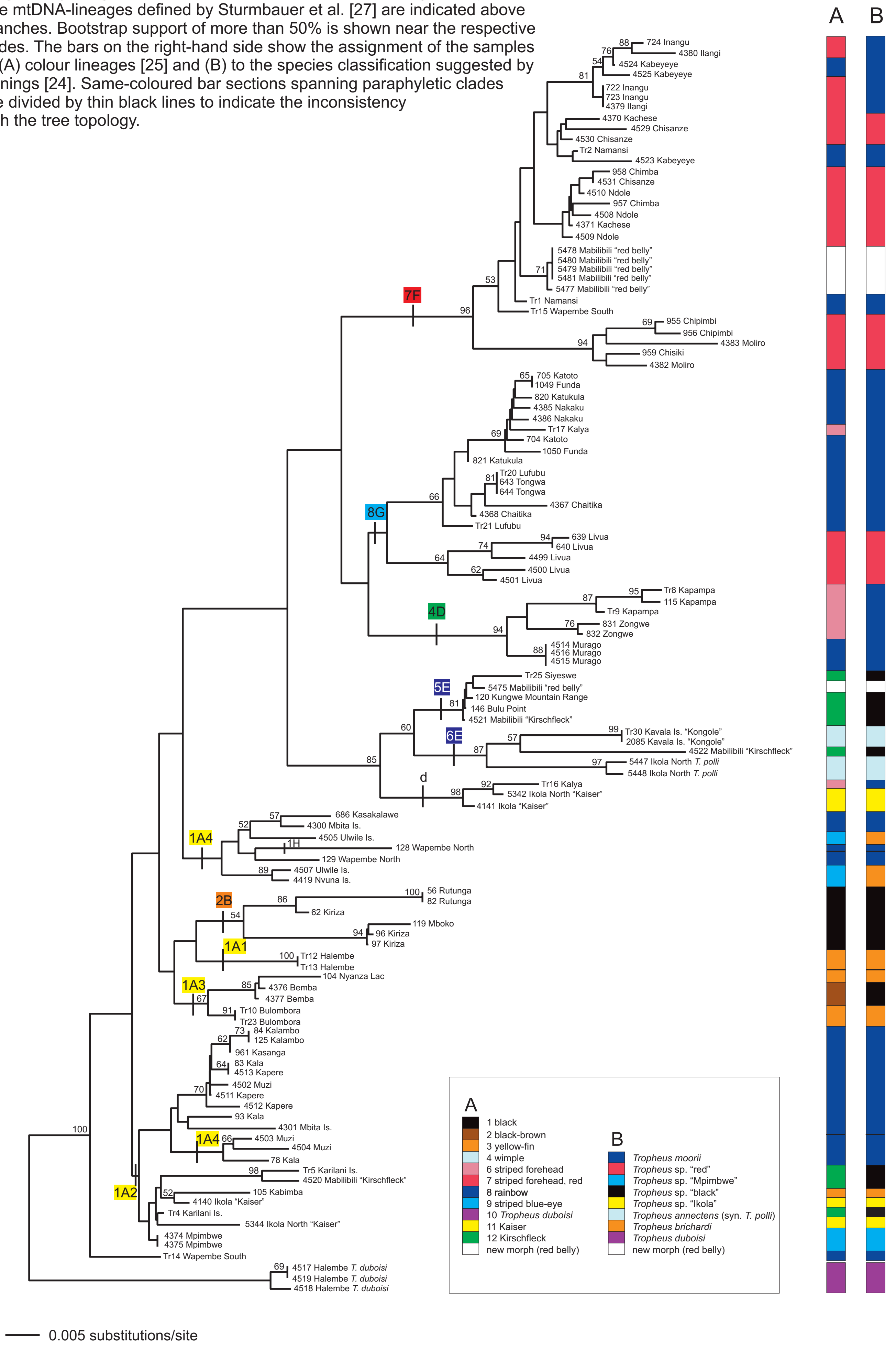

Supplement: Additional file 1 — NJ tree. NJ tree based on the mitochondrial control region sequences. [file 1471-2148-7-137-S1.pdf]
