# Supplementary material for: Nuclear and mitochondrial data reveal different evolutionary processes in the Lake Tanganyika cichlid genus Tropheus
Source: BMC Evol Biol. 2007 Aug 14;7:137. doi: 10.1186/1471-2148-7-137 (PMC2000897; doi:10.1186/1471-2148-7-137)
Supplement: Additional file 2 — Detailed information on mitochondrial sequence relationships and signals of introgression and hybridisation. The file provides additional, detailed information on mitochondrial sequence relationships and signals of introgression and hybridisation. [file 1471-2148-7-137-S2.doc]

**Additional file 2 - detailed information on mitochondrial sequence relationships (1.) and signals of introgression and hybridisation (2.)**

## 1. Mitochondrial sequence relationships – positioning of samples not included in previous phylogenetic reconstructions of the genus *Tropheus*

Fifty-nine of the samples presented here were not included in the dataset of Sturmbauer et al. [27] and were positioned as follows (see Fig. 2). Individuals from Ndole, Chisanze, Kabeyeye and Ilangi in the southwestern part of the lake grouped in mtDNA-lineage 7F together with other samples from the same area. *Tropheus* from the southeastern shore (Kapere and Muzi) grouped in mtDNA-lineage 1A2, and other individuals from Muzi in 1A4; both lineages co-occur along the entire southeastern lakeshore [27]. *T.* “Ikola” from Ikola North fell into mtDNA-lineages “d” and 1A2, both of which were previously detected in samples from this area. The sympatric *T.* *polli*, also sampled from Ikola North, grouped in mtDNA-lineage 6E, which also includes the morphologically similar *T.* “Kongole” from Kavala Island (further north on the opposite lakeshore) and *T.* “Kirschfleck” from Mabilibili in the Kungwe Mountain range. The remaining *T.* “Kirschfleck” individuals from Mabilibili were placed in mtDNA-lineage 5E, a lineage only found in the Kungwe mountain range, and the more widespread mtDNA-lineage 1A2. Individuals from another morph collected at Mabilibili, the recently described *Tropheus* “red belly”, were split up into mtDNA-lineage 7F, else found on the opposite shore, and mtDNA-lineage 5E, found along the Kungwe mountain range.

## 2. Signals of introgression and hybridisation: AFLP homoplasy excess test – selected examples

A newly described morph: *Tropheus* “red belly”

This colour morph was only recently discovered by the aquarium trade [25], and is found in sympatry with *T.* “Kirschfleck” and *T*. *polli* along the Kungwe mountain range. Our samples cluster with the *T.* “Kirschfleck” and *T.* “Kaiser” morph from the central eastern shoreline and other yellow-, orange- or red-banded morphs from the northwestern lakeshore in the AFLP tree (clade AFLP 3). Removal of the “red belly” individuals resulted in a rise of the bootstrap value at node 8a2 from 418 to 948 (Fig. 3 and 4A), which strengthens support for nuclear genetic cohesion of this - phenotypically plausible - group. Note that a certain increase in bootstrap support of node 8a2 is expected upon removal of the sister clade containing the “red belly” samples (Fig. 3). However, a strong increase in bootstrap support from 64 to 96% for the group was also observed in an analogous parsimony analysis, which clustered the “red belly” samples within the “Kirschfleck” and “Kaiser” clade (data not shown). Bootstrap values at nodes 6 and 7 also increased substantially after removal of the “red bellies” (from 455 to 649 and from 719 to 907, respectively; Fig. 4A). Our data suggest that the “red bellies” represent a hybrid morph with one parent in the AFLP-3 clade – likely a *T*. “Kirschfleck” –, but the AFLP data failed to pinpoint the second candidate parent clade.

Based on mitochondrial sequences, the “red bellies” were placed in mtDNA-lineages 6E and 7F. The latter comprises mainly the red morphs, but also includes individuals of other morphs such as “Kirschfleck” from Siyeswe [27]. All other “Kirschfleck” samples grouped in mtDNA-lineage 6E together with *T. polli*, as well as in mtDNA-lineage 1A, indicating a very complex scenario where potential parental populations carry several mitochondrial haplotypes.

Yellow coloured morphs from Lufubu, Ilangi, Inangu and Kabeyeye

North of the Lufubu River, the localities Kabeyeye, Inangu and Ilangi are inhabited by morphs that display a yellowish body colouration. These populations grouped in mtDNA-lineage 7F together with red morphs from further north. The “Lufubu” samples used in this study were collected near a village called “Lufubu” located south of the river mouth, and grouped in mtDNA-lineage 8G with the bluish morphs from further south (Fig. 2). In the AFLP tree, the fish from Inangu, Kabeyeye and Lufubu were placed into the “bluish” clade (node 1a in Fig. 3), whereas individuals from Ilangi formed a sister clade to the clade comprising bluish and yellow-blotched morphs (node 2). Removal of the yellow morphs from the AFLP analysis (Fig. 4B) increased the bootstrap values at node 1 (“bluish” + “yellow-blotched”, from 154 to 666), node 1a (”bluish”, from 14 to 240), node 2 (from 219 to 776) and node 4a (“red”, from 266 to 427), and slightly raised support at node 1b (”yellow-blotched”, from 518 to 621). These homoplasy signals together with mitochondrial haplotype composition of the yellow morphs suggest ancient introgression between the red and bluish morphs, or perhaps even indicate the origin of the yellow morphs from hybridisation of these two morphs.

Red coloured *Tropheus* from Livua and Chisanze

Red *Tropheus* from Livua represent another set of samples causing substantial conflict between mtDNA and nuclear data. AFLP data placed the Livua samples as a sister clade to the other red morphs, whereas by mtDNA data, individuals from Livua formed a sister clade to the bluish morphs (mtDNA-lineage 8G). The removal of individuals from Livua resulted in a rise of bootstrap values at nodes 4b (red morphs, from 588 to 885) and 5 (from 205 to 554; Fig. 4C). As Livua is the northernmost of our red morph sample and therefore is geographically quite distant from the bluish morphs, it is puzzling how mitochondrial introgression might have taken place, when no haplotypes of the “bluish” morph (mtDNA lineage 8G) were detected in any other population of red morphs between Livua and the Lufubu River. Sturmbauer et al. [27] assumed a single, ancient colonisation and introgression of fish from south of the Lufubu into the Livua population (termed “Mvua” in Sturmbauer et al, 2005; 5 haplotypes each of mtDNA lineage 7F and 8G, respectively, in the Mvua sample). AFLP data support the inferred lineage admixture.

Chisanze is the southernmost location in our coverage of the red morph, adjacent to the above discussed yellow morphs, with mitochondrial haplotypes grouping in mtDNA lineage 7F together with the other representatives of the red morphs. Removal of Chisanze samples from the AFLP dataset had strong effects on the bootstrap values of node 4a (“red”, from 266 to 453), node 4b (“red” without Livua, from 588 to 800) and node 3 (306 to 557; Fig. 4D). These data are suggestive of introgression between *Tropheus* from Chisanze and other *Tropheus* populations, perhaps the yellow or bluish morphs from further south.

*Tropheus* from Katoto at the southwestern tip of the lake

Katoto is located in the very south of Lake Tanganyika immediately west of a large sandy bay which separates the bluish and yellow-blotched morphs. The colour pattern of Katoto *Tropheus* is intermediate between the two morphs, and the population contains haplotypes from both sides of the bay (70% haplotypes of mtDNA-lineage 8G, which is found in the bluish morphs to the north of Katoto, and 30% haplotypes of the lineages 1A2 and 1A4, which are typical for the yellow-blotched populations east of the bay; [33]). It is assumed that the haplotype composition of the Katoto population originates from an admixture of the bluish and yellow-blotched populations, which was perhaps associated with historic lake level fluctuations [27, 33]. The two samples from Katoto used in this study grouped with the bluish populations by both AFLP data and mitochondrial sequences (mtDNA-lineage 8G). However, a removal of the fish from Katoto caused a slight increase of the bootstrap value at node 1b (the “yellow-blotched” clade) from 518 to 665 (Fig. 4E), which was beyond the bootstrap value fluctuations caused by removal of other taxa. Although the signal is weak, it is consistent with the notion that the Katoto population underwent large-scale introgression across the sandy bay.
