# Supplementary material for: Nuclear and mitochondrial data reveal different evolutionary processes in the Lake Tanganyika cichlid genus Tropheus
Source: BMC Evol Biol. 2007 Aug 14;7:137. doi: 10.1186/1471-2148-7-137 (PMC2000897; doi:10.1186/1471-2148-7-137)
Supplement: Additional file 3 — List of samples used for mtDNA and AFLP analyses. The file provides a list of samples used for mtDNA and AFLP analyses, with information about sequence accession numbers, coordinates of sampling sites, and assignment of samples to species and colour lineages. [file 1471-2148-7-137-S3.doc]

**Additional file 3**

List of samples used for mtDNA and AFLP analyses with sample identifiers, mtDNA sequence accession numbers (superscript numbers indicate origin of sequences: 1: this study, 2: ref. 28, 3: ref. 29, 4: ref. 27, 5: ref. 50, 6: ref. 23), coordinates of sampling sites (if known) and assignment of samples to species (as suggested by Konings [25]) and colour lineages (defined by Schupke [24]). When more than one morph / species was sampled from a location, the species / morphs are given in parentheses with the locality. For each sample, asterisks in the last two columns indicate the use of AFLP and/or mtDNA sequence data in the analyses.

| **Sample** | **Accession #** | **Locality** | **Latidude (S)** | **Longitude (E)** | **Species (after Konings 1998)** | **Colour lineage (after Schupke 2003)** | **AFLP** | **mtDNA** |
| --- | --- | --- | --- | --- | --- | --- | --- | --- |
| 4376 | EF694249^1^ | Bemba | 03°37' | 29°09' | *Tropheus* sp. "black" | 2 Blackbrown | * | * |
| 4377 | EF694250^1^ | Bemba | 03°37' | 29°09' | *Tropheus* sp. "black" | 2 Blackbrown | * | * |
| Tr10 | AY660826^4^ | Bulombora | 05°02' | 29°46' | *Tropheus brichardi* | 3 Yellow Fins | * | * |
| Tr23 | AY660827^4^ | Bulombora | 05°02' | 29°46' | *Tropheus brichardi* | 3 Yellow Fins | * | * |
| 146 | Z12090^2^ | Bulu Point | 06°01' | 29°45' | *Tropheus* sp. "black" | 12 Kirschfleck | * | * |
| 4367 | EF694243^1^ | Chaitika | 08°34' | 30°47' | *Tropheus moorii* | 8 Rainbow | * | * |
| 4368 | EF694244^1^ | Chaitika | 08°34' | 30°47' | *Tropheus moorii* | 8 Rainbow | * | * |
| 957 | AJ489645^3^ | Chimba | 08°19' | 30°32' | *Tropheus* sp. "red" | 7 Striped forehead, red | * | * |
| 958 | AJ489646^3^ | Chimba | 08°19' | 30°32' | *Tropheus* sp. "red" | 7 Striped forehead, red | * | * |
| 955 | AJ489643^3^ | Chipimbi | 08°17' | 30°34' | *Tropheus* sp. "red" | 7 Striped forehead, red | * | * |
| 956 | AJ489644^3^ | Chipimbi | 08°17' | 30°34' | *Tropheus* sp. "red" | 7 Striped forehead, red | * | * |
| 4529 | EF694280^1^ | Chisanze |  |  | *Tropheus* sp. "red" | 7 Striped forehead, red | * | * |
| 4530 | EF694281^1^ | Chisanze |  |  | *Tropheus* sp. "red" | 7 Striped forehead, red | * | * |
| 4531 | EF694282^1^ | Chisanze |  |  | *Tropheus* sp. "red" | 7 Striped forehead, red | * | * |
| 959 | AJ489705^3^ | Chisiki | 08°20' | 30°31' | *Tropheus* sp. "red" | 7 Striped forehead, red | * | * |
| 1049 | AJ492074^3^ | Funda | 08°46' | 30°59' | *Tropheus moorii* | 8 Rainbow | * | * |
| 1050 | AJ492077^3^ | Funda | 08°46' | 30°59' | *Tropheus moorii* | 8 Rainbow | * | * |
| Tr12 | AY660775^4^ | Halembe (*T. brichardi*) | 05°44' | 29°55' | *Tropheus brichardi* | 3 Yellow Fins | * | * |
| Tr13 | AY660776^4^ | Halembe (*T. brichardi*) | 05°44' | 29°55' | *Tropheus brichardi* | 3 Yellow Fins | * | * |
| 4517 | EF694231^1^ | Halembe (*T. duboisi*) | 05°44' | 29°55' | *Tropheus duboisi* | 10 *Tropheus duboisi* | * | * |
| 4518 | EF694232^1^ | Halembe (*T. duboisi*) | 05°44' | 29°55' | *Tropheus duboisi* | 10 *Tropheus duboisi* | * | * |
| 4519 | EF694233^1^ | Halembe (*T. duboisi*) | 05°44' | 29°55' | *Tropheus duboisi* | 10 *Tropheus duboisi* | * | * |
| 4140 | EF694235^1^ | Ikola | 06°41' | 30°22' | *Tropheus* sp. "Ikola" | 11 Kaiser | * | * |
| 4141 | EF694236^1^ | Ikola | 06°41' | 30°22' | *Tropheus* sp. "Ikola" | 11 Kaiser | * | * |
| 5342 | EF694237^1^ | Ikola North ("Kaiser") | 06°40' | 30°18' | *Tropheus* sp. "Ikola" | 11 Kaiser | * | * |
| 5344 | EF694238^1^ | Ikola North ("Kaiser") | 06°40' | 30°18' | *Tropheus* sp. "Ikola" | 11 Kaiser | * | * |
| 5447 | EF694239^1^ | Ikola North (*T. polli*) | 06°40' | 30°18' | *Tropheus annectens* (syn. *T. polli*) | 4 Whimple | * | * |
| 5448 | EF694240^1^ | Ikola North (*T .polli*) | 06°40' | 30°18' | *Tropheus annectens* (syn. *T. polli*) | 4 Whimple | * | * |
| 4379 | EF694251^1^ | Ilangi |  |  | *Tropheus moorii* | 7 Striped forehead, red | * | * |
| 4380 | EF694252^1^ | Ilangi |  |  | *Tropheus moorii* | 7 Striped forehead, red | * | * |
| 722 | AJ489707^3^ | Inangu | 08°29' | 30°41' | *Tropheus moorii* | 7 Striped forehead, red | * | * |
| 723 | AJ489708^3^ | Inangu | 08°29' | 30°41' | *Tropheus moorii* | 7 Striped forehead, red | * | * |
| 724 | AJ489633^3^ | Inangu | 08°29' | 30°41' | *Tropheus moorii* | 7 Striped forehead, red | * | * |
| 4523 | EF694277^1^ | Kabeyeye |  |  | *Tropheus moorii* | 8 Rainbow | * | * |
| 4524 | EF694278^1^ | Kabeyeye |  |  | *Tropheus moorii* | 8 Rainbow | * | * |
| 4525 | EF694279^1^ | Kabeyeye |  |  | *Tropheus moorii* | 8 Rainbow | * | * |
| 105 | Z12077^2^ | Kabimba | 05°33' | 29°20' | *Tropheus brichardi* | 3 Yellow Fins | * | * |
| 4370 | EF694245^1^ | Kachese | 08°29' | 30°28' | *Tropheus* sp. "red" | 7 Striped forehead, red | * | * |
| 4371 | EF694246^1^ | Kachese | 08°29' | 30°28' | *Tropheus* sp. "red" | 7 Striped forehead, red | * | * |
| 78 | Z12076^2^ | Kala | 08°08' | 30°58' | *Tropheus moorii* | 8 Rainbow | * | * |
| 83 | Z12075^2^ | Kala | 08°08' | 30°58' | *Tropheus moorii* | 8 Rainbow | * | * |
| 93 | Z12074^2^ | Kala | 08°08' | 30°58' | *Tropheus moorii* | 8 Rainbow | * | * |
| 125 | DQ202833^5^ | Kalambo | 08°36' | 31°11' | *Tropheus moorii* | 8 Rainbow | * | * |
| 84 | Z12073^2^ | Kalambo | 08°36' | 31°11' | *Tropheus moorii* | 8 Rainbow | * | * |
| Tr16 | AY660779^4^ | Kalya | 06°28' | 30°00' | *Tropheus moorii* | 6 Striped forehead | * | * |
| Tr17 | AY660780^4^ | Kalya | 06°28' | 30°00' | *Tropheus moorii* | 6 Striped forehead | * | * |
| Tr8 | AY660772^4^ | Kapampa | 07°30' | 30°12' | *Tropheus moorii* | 6 Striped forehead | * | * |
| Tr9 | AY660773^4^ | Kapampa | 07°30' | 30°12' | *Tropheus moorii* | 6 Striped forehead | * | * |
| 115 | Z12055^2^ | Kapampa | 07°30' | 30°12' | *Tropheus moorii* | 6 Striped forehead | * | * |
| 4511 | EF694268^1^ | Kapere |  |  | *Tropheus moorii* | 8 Rainbow | * | * |
| 4512 | EF694269^1^ | Kapere |  |  | *Tropheus moorii* | 8 Rainbow | * | * |
| 4513 | EF694270^1^ | Kapere |  |  | *Tropheus moorii* | 8 Rainbow | * | * |
| Tr4 | AY660768^4^ | Karilani Island | 05°58' | 29°47' | *Tropheus* sp. "black" | 12 Kirschfleck | * | * |
| Tr5 | AY660769^4^ | Karilani Island | 05°58' | 29°47' | *Tropheus* sp. "black" | 12 Kirschfleck | * | * |
| 686 | AJ492012^3^ | Kasakalawe | 08°47' | 31°04' | *Tropheus moorii* | 8 Rainbow | * | * |
| 961 | AJ489686^3^ | Kasanga | 08°26' | 31°08' | *Tropheus moorii* | 8 Rainbow | * | * |
| 704 | AJ492038^3^ | Katoto | 08°48' | 31°01' | *Tropheus moorii* | 8 Rainbow | * | * |
| 705 | AJ492076^3^ | Katoto | 08°48' | 31°01' | *Tropheus moorii* | 8 Rainbow | * | * |
| 820 | AJ492070^3^ | Katukula | 08°43' | 30°57' | *Tropheus moorii* | 8 Rainbow | * | * |
| 821 | AJ295913^3^ | Katukula | 08°43' | 30°57' | *Tropheus moorii* | 8 Rainbow | * | * |
| Tr30 | AY660836^4^ | Kavala Island | 05°38' | 29°24' | *Tropheus annectens* (syn. *T. polli*) | 4 Whimple | * | * |
| 2085 | AY660830^4^ | Kavala Island | 05°38' | 29°24' | *Tropheus annectens* (syn. *T. polli*) | 4 Whimple | * | * |
| 62 | Z12069^2^ | Kiriza | 04°03' | 29°13' | *Tropheus* sp. "black" | 1 Black | * | * |
| 96 | EF694234^1^ | Kiriza | 04°03' | 29°13' | *Tropheus* sp. "black" | 1 Black | * | * |
| 97 | AY929978^6^ | Kiriza | 04°03' | 29°13' | *Tropheus* sp. "black" | 1 Black | * | * |
| 120 | Z12087^2^ | Kungwe Mountain Range | 06°01' | 29°45' | *Tropheus* sp. "black" | 12 Kirschfleck | * | * |
| 639 | AJ489649^3^ | Livua | 08°05' | 30°32' | *Tropheus* sp. "red" | 7 Striped forehead, red |  | * |
| 640 | AJ489650^3^ | Livua | 08°05' | 30°32' | *Tropheus* sp. "red" | 7 Striped forehead, red |  | * |
| 4417 |  | Livua | 08°05' | 30°32' | *Tropheus* sp. "red" | 7 Striped forehead, red | * |  |
| 4418 |  | Livua | 08°05' | 30°32' | *Tropheus* sp. "red" | 7 Striped forehead, red | * |  |
| 4499 | EF694257^1^ | Livua | 08°05' | 30°32' | *Tropheus* sp. "red" | 7 Striped forehead, red | * | * |
| 4500 | EF694258^1^ | Livua | 08°05' | 30°32' | *Tropheus* sp. "red" | 7 Striped forehead, red | * | * |
| 4501 | EF694259^1^ | Livua | 08°05' | 30°32' | *Tropheus* sp. "red" | 7 Striped forehead, red | * | * |
| Tr20 | AY660783^4^ | Lufubu | 08°32' | 30°44' | *Tropheus moorii* | 8 Rainbow | * | * |
| Tr21 | AY660784^4^ | Lufubu | 08°32' | 30°44' | *Tropheus moorii* | 8 Rainbow | * | * |
| 4520 | EF694274^1^ | Mabilibili ("Kirschfleck") |  |  | *Tropheus* sp. "black" | 12 Kirschfleck | * | * |
| 4521 | EF694275^1^ | Mabilibili ("Kirschfleck") |  |  | *Tropheus* sp. "black" | 12 Kirschfleck | * | * |
| 4522 | EF694276^1^ | Mabilibili ("Kirschfleck") |  |  | *Tropheus* sp. "black" | 12 Kirschfleck | * | * |
| 5475 | EF694283^1^ | Mabilibili ("red belly") |  |  | new morph | new morph | * | * |
| 5477 | EF694284^1^ | Mabilibili ("red belly") |  |  | new morph | new morph | * | * |
| 5478 | EF694285^1^ | Mabilibili ("red belly") |  |  | new morph | new morph | * | * |
| 5479 | EF694286^1^ | Mabilibili ("red belly") |  |  | new morph | new morph | * | * |
| 5480 | EF694287^1^ | Mabilibili ("red belly") |  |  | new morph | new morph | * | * |
| 5481 | EF694288^1^ | Mabilibili ("red belly") |  |  | new morph | new morph | * | * |
| 4300 | EF694241^1^ | Mbita Island | 08°46' | 31°06' | *Tropheus moorii* | 8 Rainbow | * | * |
| 4301 | EF694242^1^ | Mbita Island | 08°46' | 31°06' | *Tropheus moorii* | 8 Rainbow | * | * |
| 119 | AY660763^4^ | Mboko | 03°55' | 29°05' | *Tropheus* sp. "black" | 1 Black | * | * |
| 4382 | EF694253^1^ | Moliro | 08°12' | 30°34' | *Tropheus* sp. "red" | 7 Striped forehead, red | * | * |
| 4383 | EF694254^1^ | Moliro | 08°12' | 30°34' | *Tropheus* sp. "red" | 7 Striped forehead, red | * | * |
| 4374 | EF694247^1^ | Mpimbwe | 07°08' | 30°30' | *Tropheus* sp. "Mpimbwe" | 9 Striped Blueeyes | * | * |
| 4375 | EF694248^1^ | Mpimbwe | 07°08' | 30°30' | *Tropheus* sp. "Mpimbwe" | 9 Striped Blueeyes | * | * |
| 4514 | EF694271^1^ | Murago | 07°03' | 29°47' | *Tropheus moorii* | 8 Rainbow | * | * |
| 4515 | EF694272^1^ | Murago | 07°03' | 29°47' | *Tropheus moorii* | 8 Rainbow | * | * |
| 4516 | EF694273^1^ | Murago | 07°03' | 29°47' | *Tropheus moorii* | 8 Rainbow | * | * |
| 4502 | EF694260^1^ | Muzi |  |  | *Tropheus moorii* | 8 Rainbow | * | * |
| 4503 | EF694261^1^ | Muzi |  |  | *Tropheus moorii* | 8 Rainbow | * | * |
| 4504 | EF694262^1^ | Muzi |  |  | *Tropheus moorii* | 8 Rainbow | * | * |
| 4385 | EF694255^1^ | Nakaku | 08°40' | 30°54' | *Tropheus moorii* | 8 Rainbow | * | * |
| 4386 | EF694256^1^ | Nakaku | 08°40' | 30°54' | *Tropheus moorii* | 8 Rainbow | * | * |
| Tr1 | AY660765^4^ | Namansi | 07°32' | 30°36' | *Tropheus moorii* | 8 Rainbow | * | * |
| Tr2 | AY660766^4^ | Namansi | 07°32' | 30°36' | *Tropheus moorii* | 8 Rainbow | * | * |
| 4508 | EF694265^1^ | Ndole |  |  | *Tropheus* sp. "red" | 7 Striped forehead, red | * | * |
| 4509 | EF694266^1^ | Ndole |  |  | *Tropheus* sp. "red" | 7 Striped forehead, red | * | * |
| 4510 | EF694267^1^ | Ndole |  |  | *Tropheus* sp. "red" | 7 Striped forehead, red | * | * |
| 4419 | EF694289^1^ | Nvuna Island | 07°26' | 30°33' | *Tropheus brichardi* | 9 Striped Blueeyes | * | * |
| 104 | Z12054^2^ | Nyanza Lac | 04°20' | 29°35' | *Tropheus brichardi* | 3 Yellow Fins | * | * |
| 56 | Z12050^2^ | Rutunga | 03°40' | 29°19' | *Tropheus* sp. "black" | 1 Black | * | * |
| 82 | Z12049^2^ | Rutunga | 03°40' | 29°19' | *Tropheus* sp. "black" | 1 Black | * | * |
| Tr25 | AY660786 | Siyeswe | 06°21' | 29°48' | *Tropheus* sp. "black" | 12 Kirschfleck | * | * |
| 643 | AJ492100^3^ | Tongwa | 08°40' | 30°53' | *Tropheus moorii* | 8 Rainbow | * | * |
| 644 | AJ492101^3^ | Tongwa | 08°40' | 30°53' | *Tropheus moorii* | 8 Rainbow | * | * |
| 4505 | EF694263^1^ | Ulwile Island |  |  | *Tropheus brichardi* | 9 Striped Blueeyes | * | * |
| 4507 | EF694264^1^ | Ulwile Island |  |  | *Tropheus brichardi* | 9 Striped Blueeyes | * | * |
| 128 | Z75705^2^ | Wapembe North | 07°54' | 30°49' | *Tropheus moorii* | 8 Rainbow | * | * |
| 129 | Z12048^2^ | Wapembe North | 07°54' | 30°49' | *Tropheus moorii* | 8 Rainbow | * | * |
| Tr14 | AY660777^4^ | Wapembe South | 08°00' | 30°53' | *Tropheus moorii* | 8 Rainbow | * | * |
| Tr15 | AY660778^4^ | Wapembe South | 08°00' | 30°53' | *Tropheus moorii* | 8 Rainbow | * | * |
| 4415 |  | Zongwe | 07°18' | 30°08' | *Tropheus moorii* | 6 Striped forehead | * |  |
| 4416 |  | Zongwe | 07°18' | 30°08' | *Tropheus moorii* | 6 Striped forehead | * |  |
| 831 | AJ489630^3^ | Zongwe | 07°18' | 30°08' | *Tropheus moorii* | 6 Striped forehead |  | * |
| 832 | AJ489631^3^ | Zongwe | 07°18' | 30°08' | *Tropheus moorii* | 6 Striped forehead |  | * |
